# Supplementary material for: A Critical View of the Application of the APEX Software (Aqueous Photochemistry of Environmentally-Occurring Xenobiotics) to Predict Photoreaction Kinetics in Surface Freshwaters
Source: Molecules. 2019 Dec 18;25(1):9. doi: 10.3390/molecules25010009 (PMC7017383; doi:10.3390/molecules25010009)
Supplement: Supplementary file 1 [file molecules-25-00009-s001.zip › 3.2.4_gcc-4.4.0.zip!/3.2.4_gcc-4.4.0/3.2.4_gcc-4.4.0/doc/octave/html/interpreter/Color-Conversion.html]

Color Conversion - Untitled


Previous: Plotting on top of Images,
Up: Image Processing


---

### 31.5 Color Conversion

Octave supports conversion from the RGB color system to NTSC and HSV
and vice versa.

— Function File: hsv\_map = **rgb2hsv** (rgb\_map)  
> Transform a colormap or image from the rgb space to the hsv space.
>
> A color n the RGB space consists of the red, green and blue intensities.
>
> In the HSV space each color is represented by their hue, saturation
> and value (brightness). Value gives the amount of light in the color.
> Hue describes the dominant wavelength.
> Saturation is the amount of Hue mixed into the color.
>
>
>
> **See also:** hsv2rgb.

— Function File: rgb\_map = **hsv2rgb** (hsv\_map)  
> Transform a colormap or image from the hsv space to the rgb space.
>
>
>
> **See also:** rgb2hsv.

— Function File: **rgb2ntsc** (rgb)  
> Transform a colormap or image from RGB to NTSC.
>
>
>
> **See also:** ntsc2rgb.

— Function File: **ntsc2rgb** (yiq)  
> Transform a colormap or image from NTSC to RGB.
>
>
>
> **See also:** rgb2ntsc.
